# The metabotyping of an East African cassava diversity panel: A core collection for developing biotic stress tolerance in cassava

**DOI:** 10.1371/journal.pone.0242245

**Published:** 2020-11-18

**Authors:** Laura Perez-Fons, Tatiana M. Ovalle, M. N. Maruthi, John Colvin, Luis Augusto Becerra Lopez-Lavalle, Paul D. Fraser

**Affiliations:** 1 Department of Biological Sciences, Royal Holloway University of London, Egham, United Kingdom; 2 International Center of Tropical Agriculture (CIAT) and Bioversity Alliance, Cali, Colombia; 3 Natural Resources Institute, University of Greenwich, Gillingham, Kent, United Kingdom; Graduate University of Advanced Technology, ISLAMIC REPUBLIC OF IRAN

## Abstract

Cassava will have a vital role to play, if food security is to be achieved in Sub-Saharan Africa, especially Central and East Africa. The whitefly *Bemisia tabaci* poses a major threat to cassava production by small holder farmers in part due to their role as a vector of cassava mosaic begomoviruses (CMBs) and cassava brown streak ipomoviruses (CBSIs). In the present study untargeted metabolomics has been used as a tool to assess natural variation, similarities and attempts to identify trait differentiators among an East African cassava diversity panel that displayed tolerance/resistance to the effects of *Bemisia tabaci* infestation. The metabolome captured, was represented by 1529 unique chemical features per accession. Principal component analysis (PCA) identified a 23% variation across the panel, with geographical origin/adaption the most influential classification factors. Separation based on resistance and susceptible traits to *Bemisia tabaci* could also be observed within the data and was corroborated by genotyping data. Thus the metabolomics pipeline represented an effective metabotyping approach. Agglomerative Hierarchical Clustering Analysis (HCA) of both the metabolomics and genotyping data was performed and revealed a high level of similarity between accessions. Specific differentiating features/metabolites were identified, including those potentially conferring vigour to whitefly tolerance on a constitutive manner. The implications of using these cassava varieties as parental breeding material and the future potential of incorporating more exotic donor material is discussed.

## Introduction

Plant breeding relies on the selection of accessions carrying desirable agronomic and consumer traits. This is not always a straightforward task, especially when the resolution or sensitivity of the phenotyping methodology is low or heavily influenced by environmental factors. The robustness of phenotyping criteria for assessing disease resistance in cassava has been already addressed, and has highlighted the effects of the environment on the visible symptoms of disease [1] and on the development rate of its vector, the whiteflies [2]. In the absence of reliable or clearly distinguishable phenotypes, metabolite analysis has been introduced as a tool for aiding selection and assessing effects of genetic differences, both among candidate donors (parents), siblings or even families derived from genetic crosses [3–6]. Food security is one of the major challenges facing society in the 21^st^ century and over 800 million of people rely on cassava production to subsist. Viral diseases like the cassava mosaic and cassava brown streak diseases (CMD and CBSD) constitute one of the major threats for cassava production in Sub-Saharan Africa (SSA) [7]. The presence of these viruses was first detected in Uganda and Tanzania back in the 1930s. CMD has been reported throughout SSA while CBSD is currently restricted to Eastern and Southern Africa but its predicted spread to West Africa is considered highly damaging. Outbreaks of CMD were controlled in the past through integrated pest management (IPM) measures including the release of advanced varieties bred for disease resistance [1, 8] but those were susceptible to CBSD. This together with the “superabundant” populations of African cassava whitefly *Bemisia tabaci* in the 2000s [9] caused new CBSD outbreaks [10]. African cassava whitefly, *Bemisia tabaci* (Gennadius) *sensu lato* is the vector of both viral agents causing CMD and CBSD but it also affects crop production directly, through the feeding of adults and nymphs, which in turn promotes the colonisation of sooty mould fungus on leaves. Since the raise and spread of African cassava whitefly superabundant populations, the development of improved cassava varieties possessing virus and whitefly resistance has constituted one of the core milestones of national breeding programs in Sub-Saharan African countries and international consortiums [8, 11, 12]. Efforts in finding natural sources of African whitefly (*Bemisia tabaci*) resistance have been focused on collections of local landraces and advanced lines originally developed for virus resistance [13–17] with the intention of pyramiding both disease and vector resistance into farmer-preferred varieties adapted to local agro-ecosystems.

In order to increase agricultural production and combat food (and nutritional) security, new crop varieties are urgently required [18]. In the case of cassava, varieties displaying tolerance to biotic and abiotic stresses, while maintaining consumer quality will be vital. To develop such lines, new alleles or combinations from “exotic” diverse germplasm need to be introgressed into existing donor or domesticated germplasm. In the present article we have used metabolomics as a phenotyping tool (metabotyping), in conjunction with genotyping by sequencing to characterise elite and pre-breeding materials found among cassava varieties presently used in East Africa. These materials are commonly referred to as the “5CP” material due to their assembly in the 5CP project [14]. Despite their importance as core resources for the development of new cassava varieties, very little detail is known on their genetic pedigree or chemical composition. The present study has provided valuable insights into these criteria that underpin their use in further plant breeding activities.

## Materials and methods

### Plant material

The clean and virus-free plant material used in this study originated from the 5CP project [14]. Plants were grown in a mixture of soil and compost (John Innes No.2; Fargo Ltd., Arundel, UK) in an insect-free quarantine glasshouse at 25 ± 5°C, 50–60% relative humidity and L14:D10 (light:dark) hours for three months. The 5CP´s cassava collection of elite varieties (Table 1) were grown under identical greenhouse conditions in order to minimise environmental effects and assess natural chemical variation under controlled conditions. Between 3 and 6 biological replicates per variety were collected when possible and metabolite composition analysed.

**Table 1:** Descriptive metadata of the East African elite cassava collection recovered from literature surveillance.

				Classification based on plant’s reaction to disease		Classification based on symptoms severity and virus titre
Country of origin	Code	Common name	*B*. *tabaci* phenotype	CMD phenotype	CBSD phenotype	African CBSVs phenotype
**Kenya**	KE1	LM1/2008/363		T [14, 17]	T [14, 17]	S [27]
	KE2	F19-NL		T [14, 17]	T [14, 17]	S [27]
	KE3	Tajirika		T [14, 17]	T [14, 17]	S [27]
	KE4	Shibe	16.5 [21]^+^	T [14, 17]	T [14, 17]	HS [27]
	KE5	F10-30-R2	16.9 [21]^+^	T [14, 17]	T [14, 17]	HS [27]
	KE6	Kibandameno		S [14, 17]	S [14, 17]	S [27]
**Malawi**	MAL1	Yizaso		T [14, 17]	T [14, 17]	HS [27]
	MAL2	Mbundumali		S [14, 17]	S [14, 17]	S [27]
		(= Manyokola [28])				
	MAL3	Sauti	15.6 [21]^+^	T [14, 17]	T [14, 17]	HS [27]
		(= CH92/077 [28])				
	MAL4	CH05/203	14.1 [21]^+^	T [14, 17]	T [14, 17]	S [27]
	MAL5	Sagonja	28.3 [21]^+^	T [14, 17]	T [14, 17]	HS [27]
	MAL6	Kalawe	25.1 [21]^+^	T [14, 17]	T [14, 17]	S [27]
**Mozambique**	MOZ1	Oekhumelela		T [14, 17]	T [14, 17]	HS [27]
	MOZ2	Eyope	25.9 [21]^+^	T [14, 17]	T [14, 17]	HS [27]
	MOZ3	Nziva		S [14, 17]	T [14, 17]	S [27]
	MOZ4	Colicanana		S [14, 17]	T [14, 17]	S [27]
	MOZ5	Orera	18.9 [21]^+^	S [14, 17]	T [14, 17]	S [27]
**Tanzania**	TZ1	KBH 2002/ 066	27.2 [21]^+^	T [14, 17]	T [14, 17]	HS [27]
		(= Kipusa [27])				
	TZ2	Pwani		T [14, 17]	T [14, 17]	S [27]
	TZ3	Mkumba	9.4 [21]^+^	S [14, 17]	T [14, 17]	S [27]
	TZ4	Kizimbani		T [14, 17]	T [14, 17]	HS [27]
	TZ5	KBH 2006/ 26	10.3 [21]^+^	T [14, 17]	T [14, 17]	HS [27]
		(= Mkuranga1 [27])				
	TZ6	Albert	12.5 [21]^+^	R [14, 17]	S [14, 17]	HS [30], S [27]
			WF-S [29]			
	TZ7	Kiroba		S [14, 17]	T [14, 17]	S [27, 30]
	TZ8	Mkombozi	20.4 [21]^+^	R [14, 17]	S [14, 17]	S [27]
		(= MM 96/4684 [28])				
**Uganda**	UG1	Nase 3	16 [21]^+^	T [14, 17]	T [14, 17]	S [27]
		(= TMS 30572 [16], Migyera, Nicass 1 [28])	WF-S [15, 16]			
	UG2	TME 204	WF-M [13]**	R [14, 31]	S [14, 31]	HS [27, 30]
	UG3	Tz 130	11.6 [21]^+^	R [14, 17, 31]	T [14, 17, 31]	HS [27], S [30]
			WF-R [15]			
		(= NaroCass1, NAM130, MM2006/130*)	WF-M [13]**			
	UG4	Nase 18	12.5 [21]^+^	R [14, 17]	T [14, 17]	HS [27]
	UG5	72-TME 14	WF-M [13]**	R [31]	S [17, 31]	HS [27]
		(= Nase 19)				
	UG6	Nase 14	20.7 [21]^+^	R [14, 17, 31]	T [14, 17, 31]	HS [27], S [30]
		(= MM 96/4271, also bread as MM 192/0248 [32])	WF-R [16]			
	UG7	Nase 1		R [14, 17]	T [14, 17]	S [27, 30]
		(= TMS60142 [11])				

HS, highly susceptible; S, susceptible; T, tolerant; R, resistant. ^*^Breeding line MM2006/130 nominated as Naro-Cass 1 and Naro-Cass 2 in the same reference [8]. [NARO = National Agricultural Research Organisation in Uganda]. ^**^Tested for whitefly phenotype in ref.9 but results not reported for this line other than it doesn't fall into the 10 top resistant nor into the 10 top susceptible. WF-M stands for whitefly-moderate phenotype. + B. tabaci abundance as in ref. [21].

### Sample collection and preparation

The 4th full expanded leaf of 3 months old plants was collected and used for analysis. Leaves collected were immediately frozen in dry ice, lyophilised for 2 days and ground to a fine powder with a TissueRuptor (Qiagen). Ten milligrams of freeze-dried powdered leaves were used for extraction of metabolites and a quality control (QC) sample was prepared by pooling 10 mg of each variety and biological replicate.

### Metabolites’ extraction

Addition of methanol:water:chloroform (1:1:2) (vol/vol/vol) to 10 mg of freeze-dried leaf material enabled separation of semi-polar metabolites in the epiphase and non-polar metabolites in the organic phase. Solvent extraction was carried out for 1 hr at room temperature with continuous shaking and the phase separation obtained after centrifugation of this mixture. Semi-polar extracts were filtered (0.45 μm nylon membranes), centrifuged (14 000 rpm, 5 min) and an aliquot of 95 μl spiked in with 5 μl of internal standard (genistein 0.2 mg/ml), were then subjected to LC-MS analysis. Details described in [19].

### LC-MS metabolite profiling

Analysis was conducted with a UHPLC UltiMate 3000 (Dionex Softron) linked to a MAXIS UHR-Q-TOF mass spectrometer (Bruker Daltonics) and an electrospray ionisation (ESI) source operating in negative mode under the following conditions: dry gas at 8 L/min, capillary 3500 V, end plate at -500 V, vaporizing temperature was 195°C and nebulizer was 1.3 Bar. Mass spectra were recorded in full scan mode from 50 to 1200 m/z range. Chromatographic separation of metabolites was carried out on an YMC-UltraHTPro C18 2 μm column (100 x 2 mm i.d.) using 10% acetonitrile in water (A) and acetonitrile (B) as mobile phases, both containing 0.1% formic acid. These solvents were used in linear gradient mode from 100% (A) to 65% (A) in 17 min and up to 0% (A) over 12 min. A 5 min washing and re-equilibration steps respectively were added to the gradient program. The flow rate was 0.2 ml/min and the injection volume used was 5 μl.

### Metabolomics data processing and analysis

Chemical features were extracted using MetaMS script in R [20] as described in [19]. Schemes of analytical, data processing and data analysis workflows and strategies adopted for interrogating data are illustrated in S1 and S2 Figs.

### Data analysis strategy and statistical analysis

An untargeted extraction and analytical methodology was used to assess the natural variation present within the diversity panel of the 5CP cassava collection. Three multivariate tests were used to explore the untargeted data matrix: (i) unsupervised Principal Component Analysis (PCA) to check for quality of the data and to obtain a general overview of the trends within the data, (ii) Agglomerative Hierarchical Clustering Analysis (HCA) to ascertain similarities and distance between varieties, and (iii) supervised classification modelling using Orthogonal Partial Least Square-Discriminant Analysis (OPLS-DA) to evaluate robustness and accuracy of metabolite fingerprinting for explaining whitefly phenotype (resistance vs. susceptibility) and at the same time enabling identification of potential biochemical markers linked to the phenotype.

An overview of the metabolome analysis was assessed by principal component analysis (PCA) and the classification models of phenotypes evaluated using OPLS-DA. Varieties Mkumba (TZ3), Albert (TZ6) and Nase 18 (UG4) as whitefly resistant (WF-R), and Sagonja (MAL5), Kalawe (MAL6) and Eyope (MOZ2) as whitefly susceptible (WF-S) were used as a subset for model prediction and classification (OPLS-DA). Selection of lines was based on the phenotyping data (number of *Bemisia tabaci* adults) published in [21]. Both multivariate analysis PCA and OPLS-DA were performed using pareto scaling and data matrix input was pre-processed by normalising against internal standard and after batch correction (QC normalisation).

Significant features identified through statistical tests were annotated using authors’ established libraries [5, 19]. Hypothesis generated from multivariate analysis were further validated by univariate statistical tests.

### Genotyping

DNA was extracted from 31 cassava leaf samples; using the CTAB-based protocol described by [22] with the following minor adjustment: DNA was obtained from powdered leaf tissues using Qiagen Tissue Lyser (Venlo, Netherlands). A standard amount of DNA (60 ng) per samples were processed using a protocol for 96 single nucleotide polymorphism (SNP) genotyping in cassava with the EP1TM system and 96.96 SNP type assays of Fluidigm® version S.01. This technique allowed simultaneously to collect both end-point and real-time data from a unique chip cell with 97% confidence. This protocol has been used previously in several studies to analyse cassava diversity and varietal identification [23–25].

Alleles for each SNP were scored as present, absent, or missing (failed to amplify); the resultant binary matrix was integrated in a.vcf file. The evaluation of true genetic duplicates was done using the NGSEP platform [26]; where, samples with a percentage of homozygous and heterozygous differences equal or lower than 3% were nominated. Finally, the genetic distance among genotypes was calculated based on the matrices of allele frequencies, and the cluster analysis was made using the agglomerative hierarchical clustering (AHC) algorithms with the Ward method. The resulting dendrogram was un-rooted and depicted using pltree function, implemented in the Cluster package v.2.1 for R program v.3.3.4 (R Development Core Team, 2017).

## Results

### The experimental approach used to metabotype the 5CP panel

Untargeted LC-MS analysis in negative mode, covering an m/z range from 50 to 1 200 generated a data-matrix of over 14 000 chemical features, after the application of MetaMS peak-picking algorithm. The raw data matrix was then filtered by excluding low abundant features not presenting an isotopic pattern. Typically, these low abundant features are close to the limit of detection and present high variability and poor reproducibility across replicates (RSD>30%). In order to reduce redundancy, chemical features corresponding to the isotopic species (^13^C, ^2^H, etc.), i.e., [M+1, 2, 3, 4] of parent ions [M] were also excluded. The final data matrix contained 1530 unique chemical features (S1 Fig), one being the internal standard. At this stage chemical features grouped under the same peak-cluster (PC) group were treated independently, and clustering of adducts, in-source fragments or multiple charged species was considered later for identification and characterisation purposes.

The final data matrix normalised against a single internal standard (SIS) and batch corrected contained 149 observations and 1529 variables (S1 File) and was used as input data for multivariate analysis and univariate statistics (S2 Fig). Within subjects biological variability was also calculated as coefficients of variation (%CV) and plotted as violin plots (S3 Fig). Generally, the biological variation of the varieties oscillated between 25 to 30%.

### The assessment of diversity at the genetic and biochemical level within the 5CP collection

**Fig 1:**
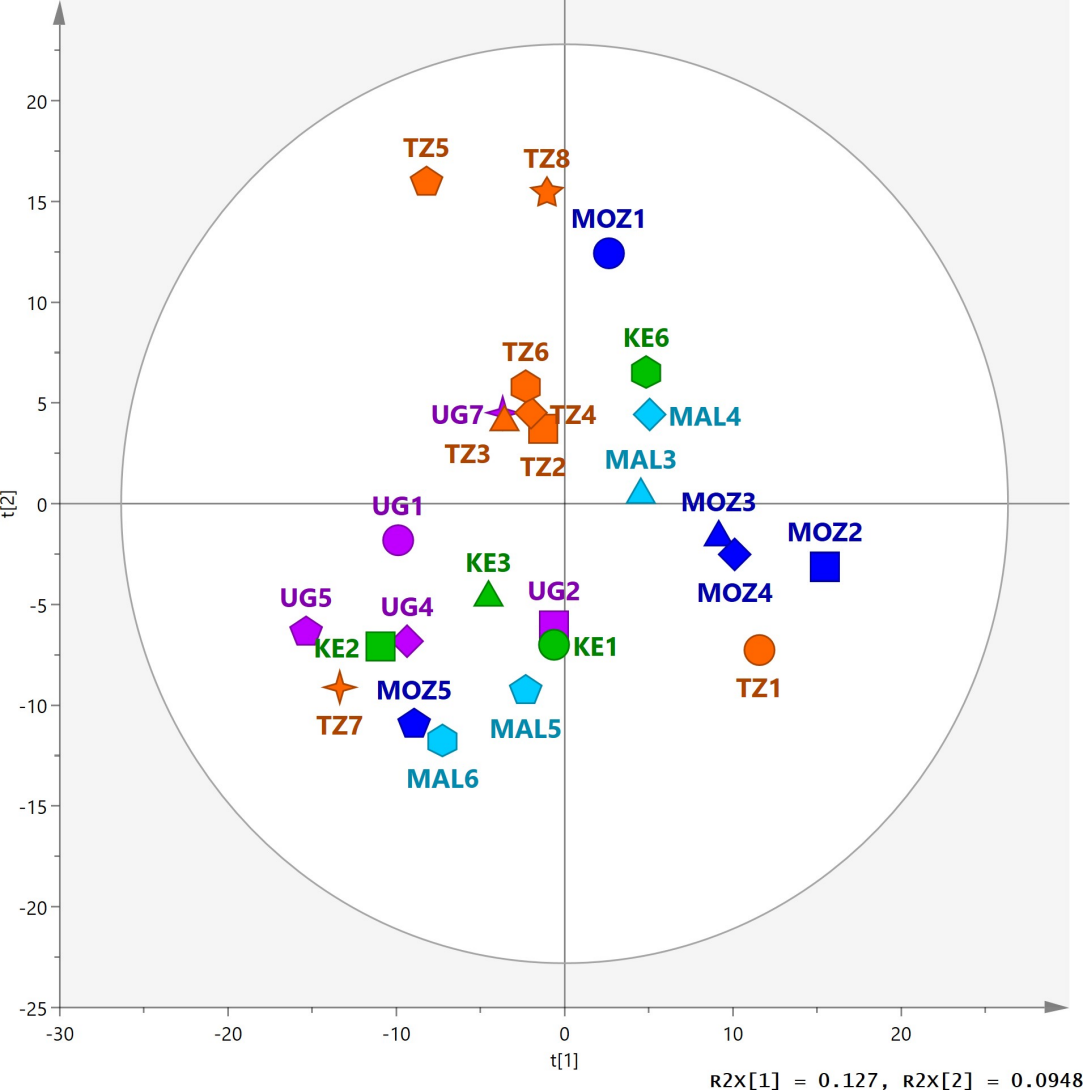
Principal component analysis of untargeted LC-MS analysis of cassava leaf extracts. Score plot of components 1 and 2 displaying median values only for visualisation purposes. Full detailed score plot illustrating biological replicates of each cassava variety is provided as supplementary material. Geographical origin is denoted with different colouring: Kenya (KE) as green, Malawi (MAL) as light blue, Mozambique (MOZ) as blue, Tanzania (TZ) as orange and Uganda (UG) as purple; and number of variety indicated as different symbols: (circle) variety 1, (box) variety 2, (triangle) variety 3, (diamond) variety 4, (pentagon) variety 5, (hexagon) variety 6, (4-point star) variety 7 and (5-point star) variety 8.

To assess the overall biochemical variation of the Eastern African cassava elite varieties, unsupervised PCA was performed on the metabolite composition of leaf extracts. Score plot of components 1 and 2 explained 23% variation of the collection which was comprised of 26 varieties (Fig 1). Three main clusters can be depicted from the PCA score plot attending geographical sub-region classification. Tanzanian varieties constituted the Central area of East Africa on the top-left sector whilst varieties collected from Northern part of the East African region, Uganda and Kenya, co-locate in the bottom-left sector of the ellipsoid. Varieties original from Mozambique and Malawi tend to cluster together and spread towards the bottom-right quadrant of the ellipsoid, especially those lines from Mozambique. Some outliers of this geographical clustering pattern are Kiroba (TZ7) and KBH2002/066 (TZ1) or Nase 1 (UG7), Kibandameno (KE6) and Oekhumelela (MOZ1). Clustering pattern associated with whitefly (resistant and susceptible) phenotypes was difficult to draw due to the insufficient data available for the complete collection. Nevertheless, Sagonja (MAL5) and Kalawe (MAL6) and Eyope (MOZ2) and KBH2002/066 (TZ1) regarded as the most susceptible by [21], cluster together in two subgroups respectively. Similarly, Tanzanian lines Mkumba (TZ3), Mkuranga 1 (KBH 2006/26, TZ5) and Albert (TZ6) described as less preferred by *B*. *tabaci* [21] form a tight cluster in the PCA score plot but separated from Nase 18 (UG4), which was also defined as whitefly resistant.

**Fig 2:**
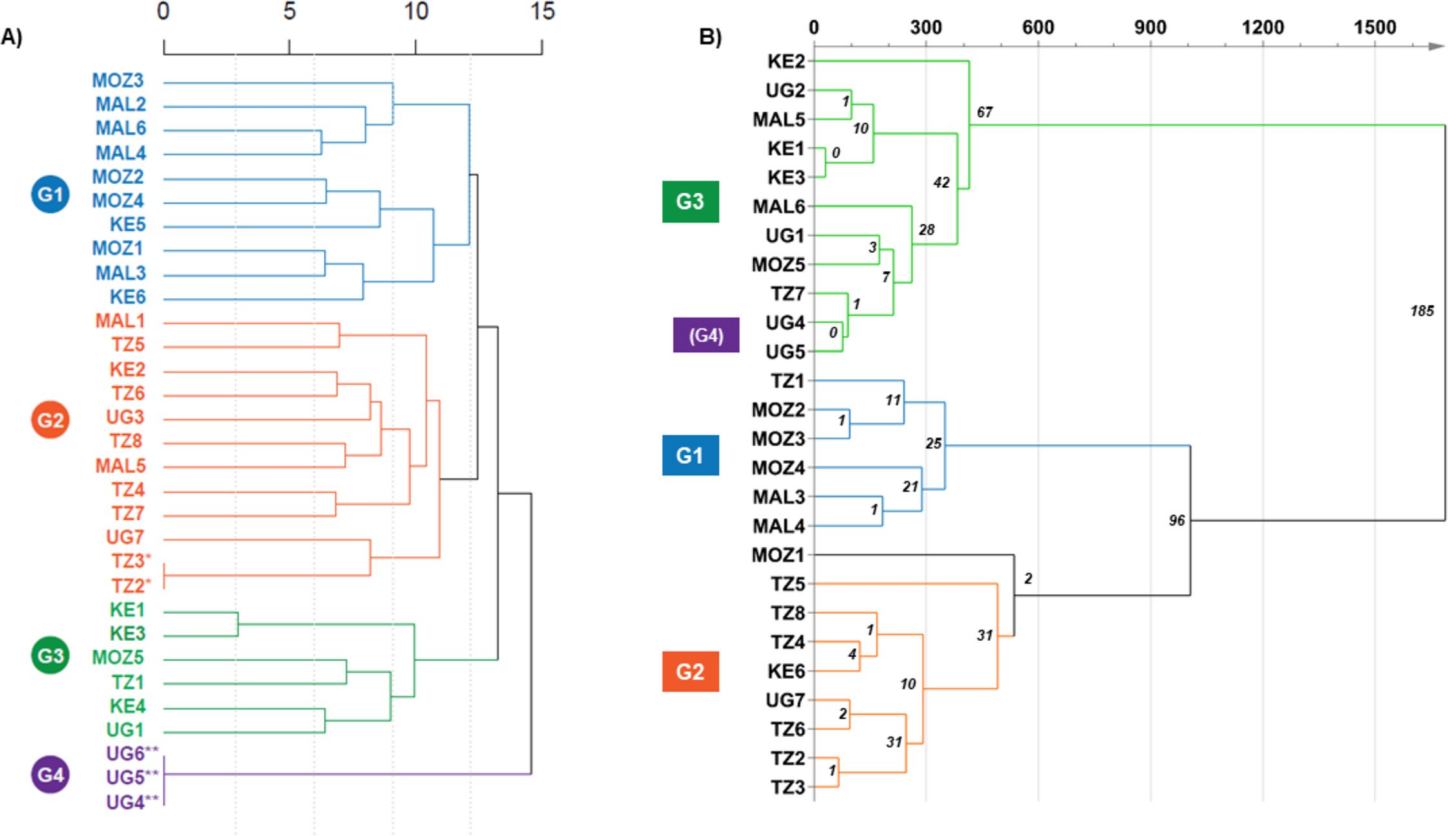
Agglomerative Hierarchical Clustering Analysis of the East African diversity panel of cassava elite varieties based on (A) genetic similarities of 96 SNP locus (n = 31 varieties) and (B) chemical features composition (n = 26 varieties) with the number of significant chemical features (multiple T-test, Holm-Sidak post-test, alfa 0.05) differentiating each metabolite-derived clustering indicated at each branching point. Kenya (KE), Malawi (MAL), Mozambique (MOZ), Tanzania (TZ) and Uganda (UG). (*) genetic duplicates within the varieties from Tanzania and (**) genetic duplicates within the varieties from Uganda.

Genetic similarity was evident when analysis of SNPs was carried out to assess the natural genetic variation of the diversity panel and compared to similarities found at chemical composition level. The Ward AHC dendrogram was constructed using the genetic similarities matrix obtained by running 96 highly informative SNP locus among the complete set of the 5CP collection, which includes 31 East African cassava genotypes (Fig 2A). These African cassava varieties clustered in four different groups; group 1 (G1) comprised principally by samples from Malawi (MAL) and Mozambique (MOZ), group 2 (G2) by samples from Tanzania (TZ), while the samples from Kenya are represented in group 3 (G3) and Uganda in group 4 (G4). The analysis of true genetic duplicates showed that among 31 African cassava genotypes, two genotypes in G2 from Tanzania, Pwani and Mkumba (TZ2 and TZ3), and three genotypes in G4 from Uganda, Nase18, 72-TME14 and Nase 14 (UG4, UG5 and UG6) showed the same genetic makeup, respectively; with zero homozygous and heterozygous differences as it is also depicted by their chemical composition. Similarly, LM1/2008/363 (KE1) and Tajirika (KE3) in group-3 clustered together based on low DNA and chemical differences (Fig 2). On the contrary, cultivars with the largest genetic and chemical differences are Tajirika (KE3) in group-3 and Oekhumelela (MOZ1) in group-1 as shown in Fig 2. It is important to highlight that G4 is not a real group; but formed due to the high number of genetic duplicated samples from Uganda. Additionally, based on both nucleotide and metabolite composition, the genotypes clustered in the same group when both analyses were compared, were Eyope (MOZ2), Nziva (MOZ3), Colicanana (MOZ4), Sauti (MAL3) and CH05/203 (MAL4) in G1, Pwani (TZ2), Mkumba (TZ3), Kizimbani (TZ4), Mkuranga 1 (TZ5), Albert (TZ6), Mkombozi (TZ8) and Nase 1 (UG7) in G2, and LM1/2008/363 (KE1), Tajirika (KE3), Orera (MOZ5), Nase 3 (UG1), Nase 18 (UG4) and 72-TME 14 (UG5) in G3 indicating a noticeable level of collinearity between the SNP information and the biochemical composition.

The number of significant chemical features differentiating each sub-group of metabolome derived dendrogram (Fig 2B) were calculated by multiple t-test comparison and indicated in the figure. The differences between the two main clusters, one comprised by G3 and G4 and the other including G1 and G2, is based on 185 significant features out of 1529 suggesting a % homology at the metabolite level of 87.9%. Similarly, the differences between groups occurring at the next branching point, G1 and G2, had a 93.8% homology, i.e. 96 significant features over a total of 1529. The percentage of similarity increases towards the base of the tree where the homology of chemical profiling between closest varieties is over 99% (Table 2).

**Table 2:** Percentage of homology at metabolite composition level based on the number of different significant chemical features within each cluster and between the most distant clusters.

			# Significant features		% Homology	
**AHC-subgroup****s**			*alfa*: *0*.01*	*alfa*:*0*.05*	*alfa*: *0*.01*	*alfa*:*0*.05*
*Sub-Group 3*.*1*	UG2	MAL5	0	1	100	99.93
*Sub-Group 3*.*2*	KE1	KE3	0	0	100	100
*Sub-Group 3*.*3*	UG1	MOZ5	2	3	99.87	99.80
*Sub-Group 3*.*4*	TZ7	UG4	0	2	100	99.87
	TZ7	UG5	0	0	100	100.00
	UG4	UG5	0	0	100	100.00
*Sub-Group 1*.*1*	MOZ2	MOZ3	2	4	99.87	99.74
*Sub-Group 1*.*2*	MAL3	MAL4	1	1	99.93	99.93
*Sub-Group 2*.*1*	TZ8	TZ4	0	1	100	99.93
	TZ8	KE6	0	3	100	99.80
	TZ4	KE6	0	4	100	99.74
*Sub-Group 2*.*1*	UG7	TZ6	1	2	99.93	99.87
*Sub-Group 2*.*2*	TZ2	TZ3	0	1	100	99.93
**Comparison of dendrogram’s extremes**			*alfa*: *0*.01*	*alfa*:*0*.05*	***alfa*: *0*.*01***	***alfa*:*0*.*05***
*sG2*.*3 vs*. *sG3*.*1*	TZ2+TZ3	UG2+MAL5	16	32	98.95	97.91
*sG2*.*3 vs*. *sG3*.*2*	TZ2+TZ3	KE1+KE3	19	43	98.75	97.19
**Comparison of WF phenotype extremes**			*alfa*: *0*.01*	*alfa*:*0*.05*	***alfa*: *0*.*01***	***alfa*:*0*.*05***
*sG2*.*3 vs*. *sG3*.*1*	TZ3 (WF-R)	MAL5 (WF-S)	1	3	99.93	99.80

*Statistical significance determined using the Holm-Sidak method, with alpha = 0.05 or 0.01. Each row (chemical feature) was analysed independently, without assuming a consistent SD. Number of t-tests: 1529.

### Biochemical features as differentiating phenotypic markers

In order to study whether the natural variation present in the collection can be utilised to define metabotypes representative of WF-R/WF-S phenotype and also to identify potential chemical markers linked to phenotype, a multivariate classification analysis was applied using the LC-MS untargeted data matrix as input.

Supervised discriminant analysis like partial least squares (PLS) are often used for these purposes. In the present study an OPLS-DA model was chosen to incorporate both the orthogonal variation which is inherent of the metabolomics mass spectrometry data and the chemical variation of the different classes of phenotypes.

**Fig 3:**
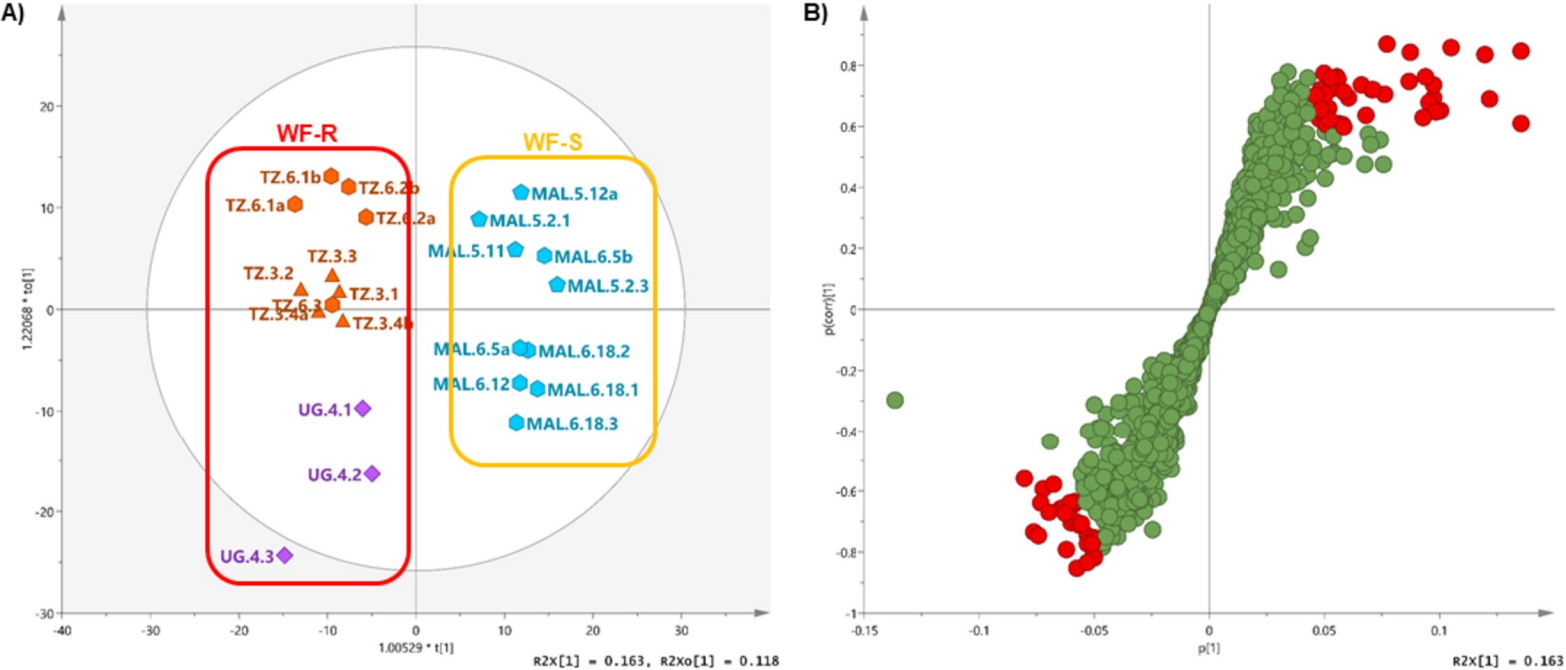
(A) Score plot of OPLS-DA of untargeted LC-MS analysis of cassava leaf extracts of whitefly resistant (WF-R) and whitefly susceptible (WF-S) classes. Geographical origin is denoted with different colouring: Malawi (MAL) as light blue, Tanzania (TZ) as orange and Uganda (UG) as purple; and number of variety indicated as different symbols: (triangle) variety 3, (diamond) variety 4, (pentagon) variety 5 and (hexagon) variety 6. (B) Loadings S-Plot with potential biomarkers (p(corr)[1]>|0.6|) and p[1]>|0.05|) highlighted in red.

Based on the whitefly abundance data available [21] OPLS-DA was applied to assess the ability of the metabolome to explain and predict the phenotype reported. The varieties presenting the most extreme phenotypes were used to build the model and extract potential biomarkers. Varieties Mkumba (TZ3), Albert (TZ6) and Nase 18 (UG4) were selected as WF-R class and Sagonja (MAL5) and Kalawe (MAL6) as WF-S. The variety Eyope (MOZ2) was initially selected and included in the WF-S but was later excluded due to the high detected variation across biological replicates (S4 Fig). Coincidentally, Sagonja (MAL5) and Kalawe (MAL6) happened to present the highest percentage of symptomatic plants when assessed for CBSD phenotype, and Mkumba (TZ3), Nase 18 (UG4) and Orera (MOZ5) displayed the lowest number of infected plants [21] thus designated as CBSD susceptible and resistant classes, respectively. The resulting model created from WF-R and WF-S classes presented strong goodness fit and prediction power (R2cum = 0.941 and Q2 = 0.762, respectively). A variability of 16.3% between classes and 11.8% within classes is explained by the model (Fig 3A) where sub-clusters can be observed within both the WF-R and WF-S class. Loadings plot in the form of S-plot (Fig 3B) was used to visualize and select the variables statistically relevant for discriminating both phenotype classes, i.e., putative biomarkers. A list of 69 potential biochemical markers was extracted based on the combined values of correlation and contribution (p(corr)[1]>|0.6|) and p[1]>|0.05|) of chemical features and ANOVA analysis was applied to additionally validate the statistical differences between the means of the different varieties (S2 File). Ten out the 69 putative markers were found to be not significant according to pair-wise comparison tests. Multiple t-test comparison to Sagonja (MAL5) as susceptible reference reveals that varieties Mkumba (TZ3) and Albert (TZ6) presented the highest number of significant differences (5 and 13 out of the 69, respectively), followed by Kalawe (MAL6) and Nase 18 (UG4) which shows 2 and 1 significant differences, respectively. This is also consistent with ANOVA’s Dunnett test using Sagonja (MAL5) as control where 40 markers out of the 69 were significantly different when compared to Mkumba (TZ3), similar number of significant features were obtained from Sagonja (MAL5)-Kalawe (MAL6) and Sagonja (MAL5)-Albert (TZ6) comparisons, 27 and 26 respectively, and the lowest number of significant differences (8 out of 69) were found between the WF-R variety Nase 18 (UG4) and the WF-S reference Sagonja (MAL5) (S2 File).

**Fig 4:**
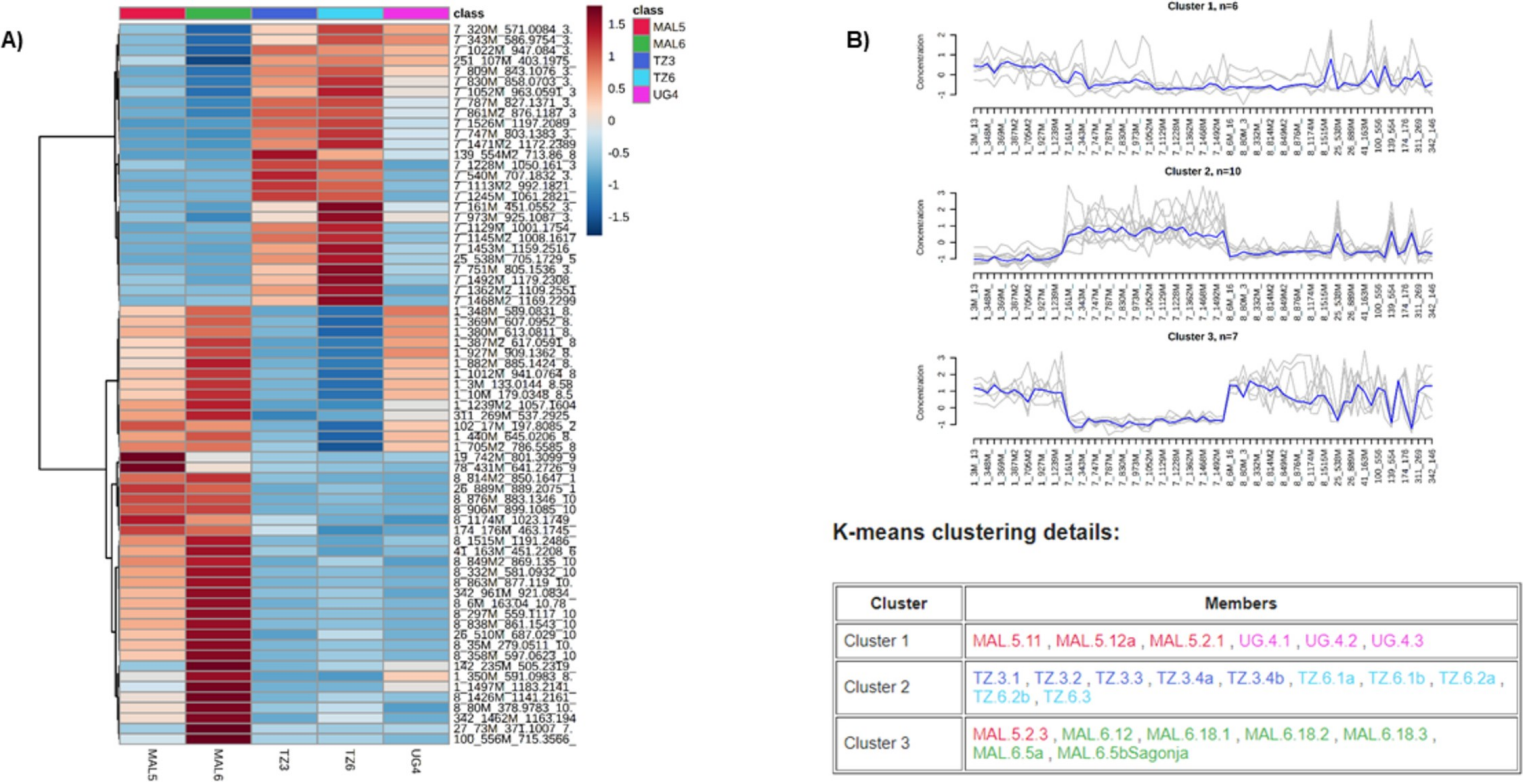
Putative markers extracted from the loadings S-plot, (A) heatmap and (B) K-means clustering of the WF-R and WF-S classes.

Representation of normalised relative concentrations of putative biomarkers across varieties in a heatmap shows that the trend in data is organised in three clusters as confirmed by K-means clustering (Fig 4). Two main clusters 2 and 3 showing reciprocal accumulation of metabolites between the WF-S varieties Sagonja (MAL5) and Kalawe (MAL6) and the WF-R varieties Mkumba (TZ3), Albert (TZ6) and Nase 18 (UG4). In cluster number 1, the WF-R line Nase 18 (UG4) presented a number of metabolites with similar levels as WF-S variety Sagonja (MAL5). Some of the chemical features identified as putative chemical markers were characterized by tandem mass spectrometry and accurate mass as malate esters of *p*-coumaric acid and caffeic acid and neochlorogenic acid (S2 File). These compounds have been previously identified to be involved in the resistance mechanism of South American cassava ECU72 challenged with the South American whitefly *Aleurotrachelus socialis* Bondar [19].

## Discussion

The natural variation at metabolite and genetic level of an East African cassava elite diversity panel is presented. Analysis of the chemical fingerprinting datasets indicated low natural variation (23%) compared to the variation found in other cassavas’ collections including both African and South American cultivars [33] where >50% and >30% was found in leaves collected in the fields and *in vitro* plantlets respectively. The high level of homology within the East African collection is consistent at both chemical and genetic level, which has been previously reported by [34]. In effect the data shows the robustness of using metabolomics as a classification tool. Capturing the representative metabolome, enables comparisons at the level of chemical composition which is often more representative of traits and more comprehensive than a limited number of DNA based markers.

Historical breeding efforts of African cassava, documented since in the 1930s [1, 8, 11, 13] suggests a high level of in-breeding probably resulting in scarce diversity in the current national stock collections and fields. Indeed, the most promising candidates within the 5CP collection, which represent a subset of national collections, as potential sources of both whitefly and disease resistance seem to be concentrated in the Ugandan and Tanzanian lines (Table 1) which are the ones presenting the highest level of duplication and homology. Although the pedigree of these candidate lines is unknown, the origins of some of the Ugandan collection can be found in the literature (S3 File), whilst Tanzanian cultivars may originate from similar ancestors, i.e., the “Amani hybrids” previously deployed to farmers and most likely bred/clonally propagated naturally over the years. Chemical distribution of East African varieties either in the PCA or dendrogram followed a geographical origin pattern and classification associated with either whitefly or virus resistance/susceptible phenotypes couldn’t be drawn. Instead a high level of similarity was found between phenotypically and even geographically distant varieties suggesting that probably the effect of adaptation to local agro-ecosystems on the plant’s metabolome dominate and mask both the whitefly and virus phenotype observed in the fields.

The variability introduced by environmental adaptation factors has likely to have an impact in defining the preference of whitefly adults for certain varieties or the symptoms developed under virus infection and therefore hindering/misleading the identification of true-to-type phenotype classes. This hypothesis/concern also arises when the list of potential biomarkers generated from the classification model (ultimately based on observed phenotype) is investigated further. Pair-wise comparison of the WF-R variety Nase 18 (UG4) and the susceptible Sagonja (MAL5) revealed higher similarity between them than comparison between Sagonja (MAL5) and Kalawe (MAL6), despite both Malawian lines being phenotyped as the most susceptible. Varieties UG4 (Nase 18) and MAL5 (Sagonja) presented a significant number of phenotypic biochemical markers displaying a similar pattern of accumulation in both lines.

A number of chemical features differentiating WF-R and WF-S classes were characterised and identified as neochlorogenic acid, caffeic acid and *p*-coumaric acid malate esters among other unknown features. Malate esters of *p*-coumaric and caffeic acid were the most influential in the S-plot and present at lower levels in the resistant varieties UG4, TZ3 and TZ6 whilst neochlorogenic acid accumulated in the Tanzanian varieties resistant to whitefly and viral diseases CMD and CBSD (S5 Fig). The identified compounds have been previously found to be also associated with the South American whitefly resistance in cassava and a mechanistic mode of action proposed [19]. The changes in the metabolite composition mapped into plant’s metabolome suggested a link between phenylpropanoids and monolignols biosynthesis and cell wall reinforcement as a resistance mechanism. The initial cassava breeding programmes witnessed the incorporation of South American germplasm, namely the Brazilian cultivars, *Aipin Valenca* and *Macaxeira aipin* (S3 File). Therefore it is potentially feasible that “ancestorial” regions of “exotic” DNA have been selected for, within these 5CP lines, that have led to the presence of the altered phenolic components (or regulators controlling their levels) that appear to contribute to vigour and resilient traits. Given the heterozygosity of the cassava, segregation of the 5CP lines from selfed accessions could lead to enhancement of the phenotype by increasing the dosage of the gene(s) of interest and/or regions influencing complex traits. However, it is important not to lose sight that the number of unknown chemical features that remain unresolved is still significant and further characterisation of these unknowns as well as implementation of further analytical metabolomics approaches are required. Annotation and characterisation of unknown molecular or chemical features is the bottleneck of untargeted metabolomics studies which limits the discovery of relevant biological markers to a reduced number of overrepresented chemical classes. Phenylpropanoids constitute one of these examples of a recurrent marker of multiple agronomic trait or biological activities with emphasis on chlorogenic acid and flavonoids [35–40]. The large-scale nature of untargeted metabolomics studies requires high throughput methods that likely favour the extraction and detection of, for example, phenylpropanoids and underestimate the presence of minor chemical families are likely to be a contributing factor to these outcomes. Although bioinformatic resources have implemented the process of interrogating metabolite and mass spectrum databases thus increasing the number of putative identifications, complementation with targeted approaches based on front-end fractionation would also aid in providing a more comprehensive coverage of plant’s metabolome diversity.

## Conclusions

This study shows that metabotyping approaches are effective methodologies for the assessment of natural variation in cassava. The corroborating genotyping emphasises the need for multi-level omic analysis to ensure robustness. Collectively, these data provide important information on parental materials that can be used in future breeding programmes directed towards cassava varieties with improved stress tolerances. The relative low level of natural variation identified in this study suggests that initial wide genetic crosses with diverse germplasm would be beneficial, prior to focussed trait specific introgression or pyramiding of poorly characterised materials. In addition, if diversity panels containing genetically unrelated germplasm can be created mGWAS is an approach that could be implemented.

## Supporting information

S1 Fig Schematic workflow followed for preparing data from raw data files to pre-processed data matrix. (TIF)

S2 Fig Strategy followed for interrogating and analysing data. (TIF)

S3 Fig (A) Table of calculated coefficient of variation (%) within biological replicates per each variety. Columns indicate median values of CV of all variables (chemical features) and the minimum and maximum CV values of each cassava variety. (B) Violin plot showing within subject coefficient of variation (biological variability) for each variety. Median values indicated as straight lines and top and bottom dashed lines indicate 75^th^ and 25^th^ quartiles respectively.
(TIF)

S4 Fig Principal component analysis of untargeted LC-MS analysis of cassava leaf extracts. Score plot of components 1 and 2 displaying all biological replicates of each cassava variety as small symbols and median values as large symbols. Geographical origin is denoted with different colouring: Kenya (KE) as green, Malawi (MAL) as light blue, Mozambique (MOZ) as blue, Tanzania (TZ) as orange and Uganda (UG) as purple; and number of variety indicated as different symbols: (circle) variety 1, (box) variety 2, (triangle) variety 3, (diamond) variety 4, (pentagon) variety 5, (hexagon) variety 6, (4-point star) variety 7 and (5-point star) variety 8.
(TIF)

S5 Fig Relative levels of most influential metabolite markers obtained from loadings S-plot comparing WF-R (UG4, TZ3, TZ6) and WF-S varieties (MAL6, MAL5). (PDF)

S1 File Matrix of raw (not filtered) and normalised data (filtered). (XLSX)

S2 File Statistics (ANOVA and t-tests) on list of potential metabolite markers obtained from OPLS-DA loadings S-plot including putative identification. (XLSX)

S3 File Scheme summarising historical breeding efforts in Africa for disease resistance in cassava crop and list of related references. (PDF)
