# Supplementary figures and images for: The metabotyping of an East African cassava diversity panel: A core collection for developing biotic stress tolerance in cassava
Source: PLoS One. 2020 Nov 18;15(11):e0242245. doi: 10.1371/journal.pone.0242245 (PMC7673516; doi:10.1371/journal.pone.0242245)

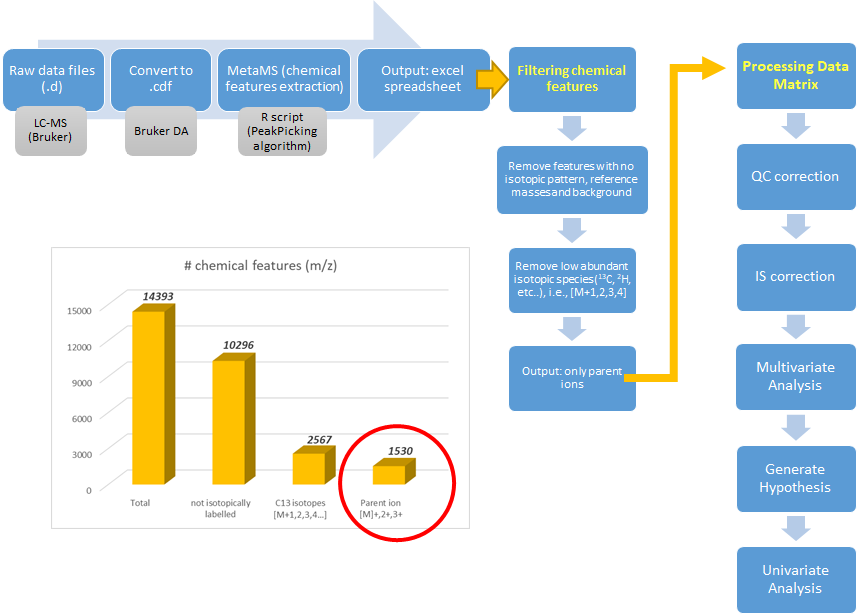

Supplement: S1 Fig — (TIF) [file pone.0242245.s001.tif]

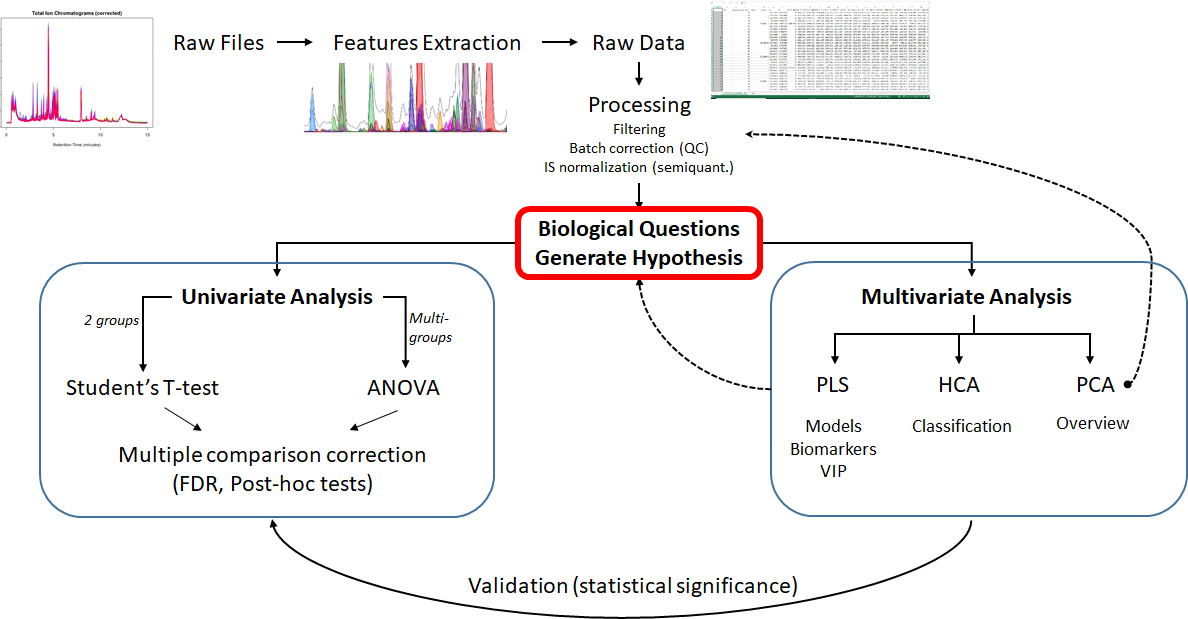

Supplement: S2 Fig — (TIF) [file pone.0242245.s002.tif]

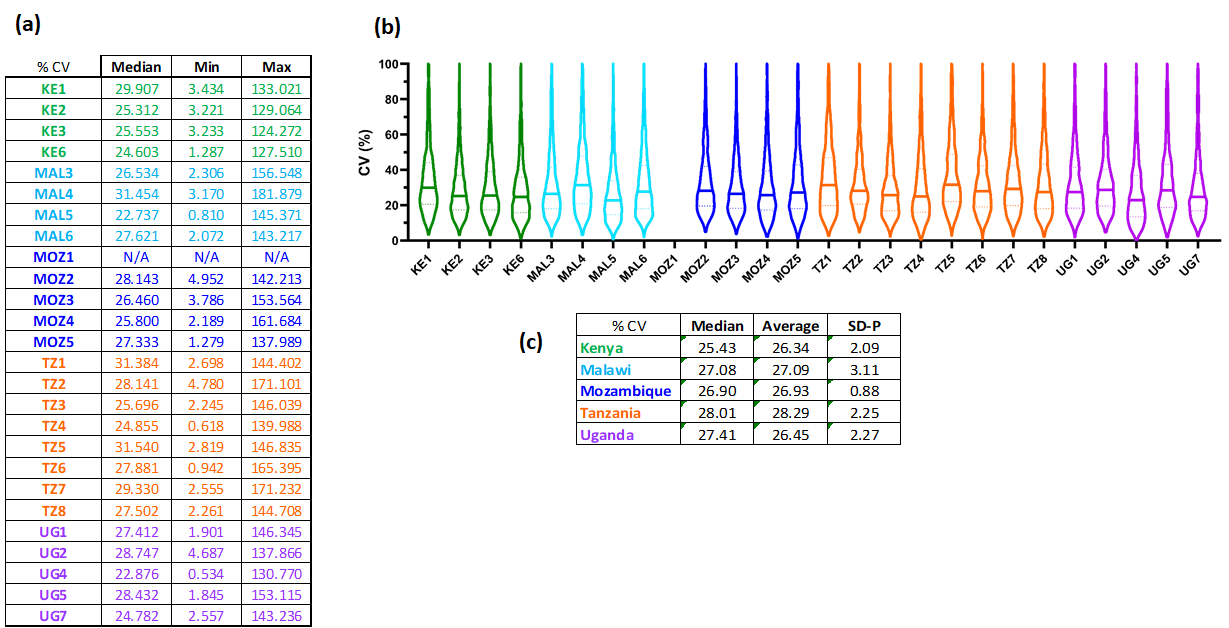

Supplement: S3 Fig — (A) Table of calculated coefficient of variation (%) within biological replicates per each variety. Columns indicate median values of CV of all variables (chemical features) and the minimum and maximum CV values of each cassava variety. (B) Violin plot showing within subject coefficient of variation (biological variability) for each variety. Median values indicated as straight lines and top and bottom dashed lines indicate 75th and 25th quartiles respectively. (TIF) [file pone.0242245.s003.tif]

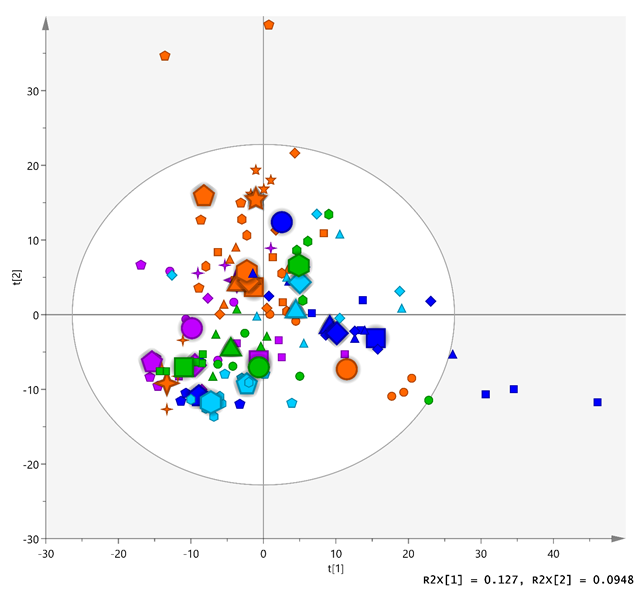

Supplement: S4 Fig — Score plot of components 1 and 2 displaying all biological replicates of each cassava variety as small symbols and median values as large symbols. Geographical origin is denoted with different colouring: Kenya (KE) as green, Malawi (MAL) as light blue, Mozambique (MOZ) as blue, Tanzania (TZ) as orange and Uganda (UG) as purple; and number of variety indicated as different symbols: (circle) variety 1, (box) variety 2, (triangle) variety 3, (diamond) variety 4, (pentagon) variety 5, (hexagon) variety 6, (4-point star) variety 7 and (5-point star) variety 8. (TIF) [file pone.0242245.s004.tif]

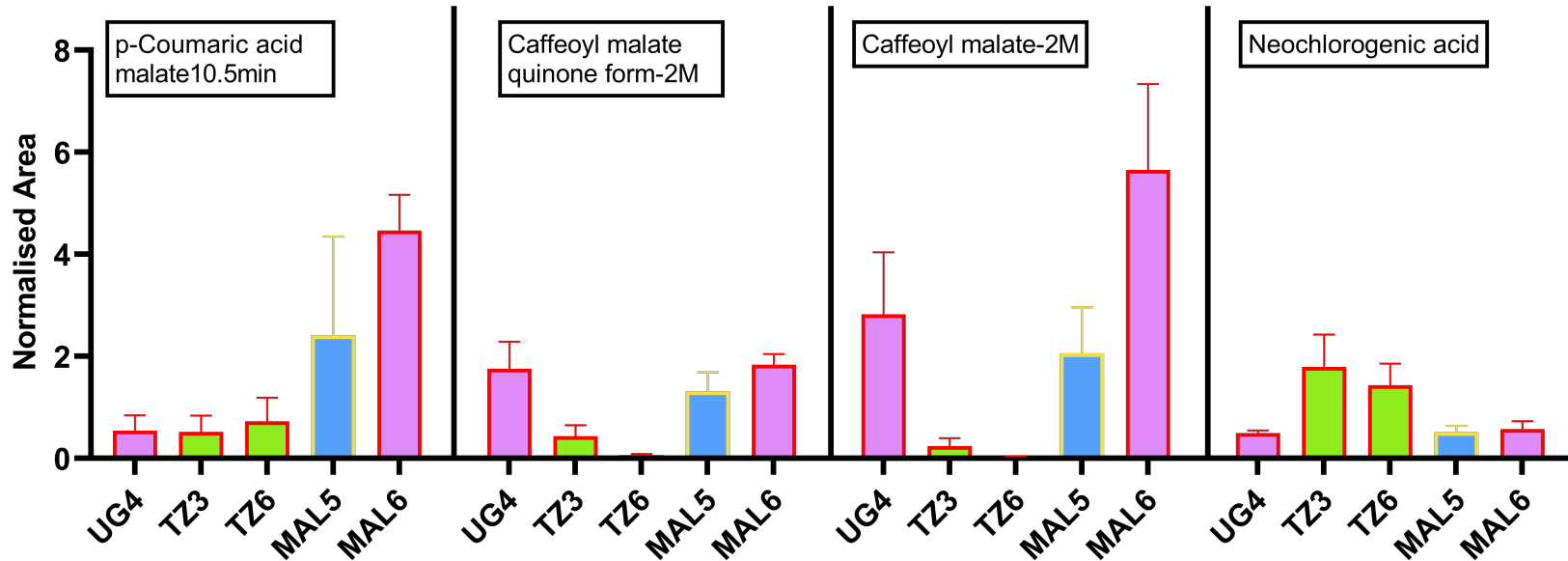

Supplement: S5 Fig — (PDF) [file pone.0242245.s005.pdf]
