# Supplementary material for: The metabotyping of an East African cassava diversity panel: A core collection for developing biotic stress tolerance in cassava
Source: PLoS One. 2020 Nov 18;15(11):e0242245. doi: 10.1371/journal.pone.0242245 (PMC7673516; doi:10.1371/journal.pone.0242245)
Supplement: S3 File — (PDF) [file pone.0242245.s008.pdf]

Historical scheme of breeding for disease resistance (CMD and CBSD) in African cassava

Metadata

***Me**: M. esculenta* ***Mg**: M. glaziovii* ***Md**: M. dichotoma* ***Mc**: M. catingae* ***Mm**: M. melanobasis (= M. esculenta subsp. flabellifolia)* ***Ms**: M. saxicola (= M. esculenta subsp. flabellifolia)*
BC: backcross
Me: Brazilian cultivars Aipin Valenca and Macaxeira aipin were used as parents for the backcrossing program as these varieties presented high CBSD resistance [Ref.13: Hillocks RJ (2003)].*
***considered as Amani hybrids too?*

Information mostly extracted from ref. 13: Hillocks RJ (2003) and complemented with additional refs.

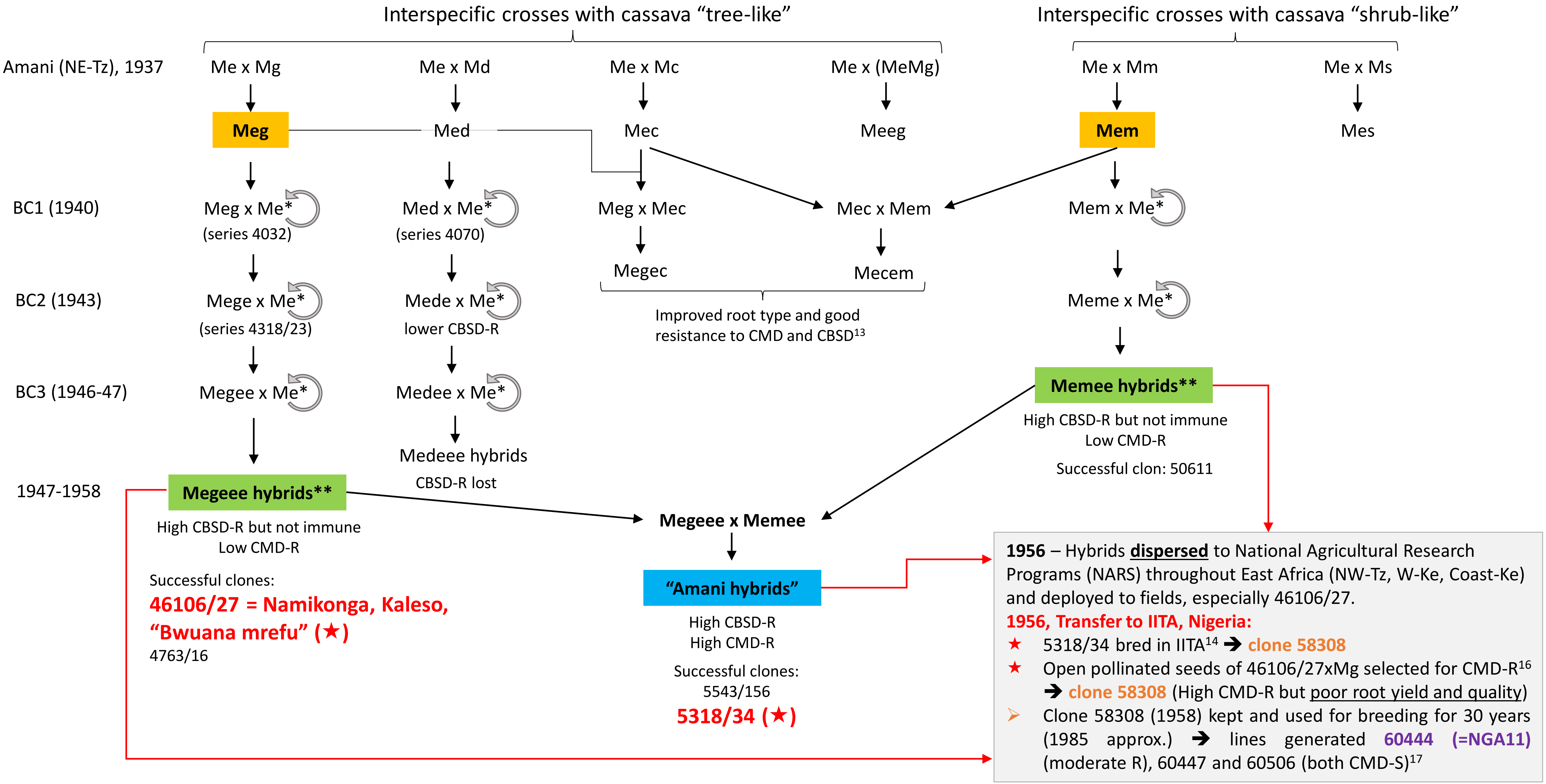

TMS = Tropical Manihot Series ***TME = Tropical Manihot Esculenta*** *OP = open-pollitaion*

IITA, Nigeria, 1971 — Breeding continued to improve CMD-R and root yield

58308 x local cultivars
58308 x Mg
Mg x local cultivars(?)

→ **TMS series**

Superior CMD-R
Increased yield

Advanced varieties released in 1977 in Nigeria:
TMS 30395
TMS 63397
TMS 30555 (=NASE 9)
TMS 4(2)1425
TMS 30572 (=NASE 3[14,15] (**UG1**))
TMS 60142 (=NASE 1[15] (**UG7**)
TMS 30337 (=NASE 2[20])

Almost immune to CMD:
TMS 30359
TMS 30001 (=Mnga-1 in India and used as CMD-R source)

→ During 1990s breeding extended to other African countries and clones with CMD-R and selected for local environment were distributed to farmers [22,23].

IITA, Nigeria, 1990s-2010s — Breeding continued to improve CMD-R by crossing sources of *CMD1* gene (TMS series) and sources of *CMD2* gene (TME collection)

TMS series x **TME collection**

Recessive CMD1 from Mg

Dominant CMD2 from Mg?

- TME is a collection of West African (mostly Nigeria) landraces presenting high levels of CMD resistance.
- TME lines are probably natural hybrids between cultivated cassava (Me) and ceara rubber (Mg) which has been growing in Nigeria for centuries[16]. They may also come from the TMS series or the Amani hybrids previously deployed to fields (?).
- Example: **TME 7, TME 14 (UG5), TME204 (UG2)**
- 1995: shared/transferred to NaCRRI, Namulonge (Uganda)

Uganda, 2004-2006 — Severe CBSD outbreak co-occurring with superabundant populations of *Bemisia tabaci*.

NaCRRI, Uganda, 2004 — Breeding for CMD and CBSD resistance using TME and TMS collections (transferred from IITA in 1995) as source of CMD-R.

TME (mostly **TME 14**) x local cultivars (Kakwele, Bamunanika[18])
TMS (mostly **TMS30555, 30572, 60142**) x local cultivars[19,22]
OP-Tanzanian (Amani?) seeds

MM and MH series

Advanced varieties released: NASE series
NASE 14 (=MM 96/4271) (**UG6**)
NASE 13 (=MH 97/2961) → CBSD-S, CMD-R
Mkombozi (**TZ8**) (=MM 96/4684)[22]
MM 2006/130 (=Naro-Cass 1[3]?) (**UG3**) → OP-Tanzanian seeds[17]
MM 06/130 (=Naro-Cass 2[21])

National Breeding Programs, 2000s — Intercrosses between local landraces[13].

Kenya: Kaleso (=46106/27), Guzo, Gushe, Kabiriti Mweusi, Ambari

Mozambique: Nikwaha, Chigoma Mafia, Nachinyaya, Xino Nn'goe, Likonde, Mulaleia, Badge

Tanzania: Namikonga (=46106/27, UG110043[24]), Kiroba (**TZ7**), Nachinyaya, Kigoma Mafia, Kitumbua, Kalulu, Mfaransa, Muzege, Gezanlole, Kibangameno (**KE6**)

References

1) Tumwegamire S (2018). Food security, 10(2): 351-368. "Exchanging and managing in-vitro elite germplasm to combat CBSD and CMD in EA and SA".
2) Maruthi MN (2019). PMPP, 105: 77-87. "A method for generating virus-free cassava plants to combat viral disease epidemics in Africa".
3) Tumwegamire S (2019), Crop Protection, 120: 58-66. "Varietal response of cassava root yield components and root necrosis from CBSD to time of harvesting in Uganda".
4) Gwuandu C et al. Afr Crop Sci J (2017), 27(2): 213-228."Whitefly resistance in African cassava genotypes"
5) Mwila N (2017). Afr Crop Sci J, 25(3): 365-385. "Biochemical factors associated with cassava resistance to whitefly infestation".
6) Omongo CA (2012). J Integr Agric, 11(2): 327-336. "African cassava whitefly, *Bemisia tabaci*, resistance in Africa and South American cassava genotypes".
7) Sheat S (2019). Front Plant Sci, 10: article 567. "Resistance against cassava brown streak viruses from Africa in cassava germplasm from South America".
8) Shirima RR (2020). Virus Res, 286 article 198017. "Genotype by environment cultivar evaluation for CBSD resistance in Tanzania".
9) Kaweesi T (2014). Vir J, 11: article 216. "Field evaluation of selected cassava genotypes for cassava brown streak disease based on symptom expression and virus load".
10) Milenovic M (2019). Front Plant Sci, 10: article 1. "Impact of host plant species and whitefly species on feeding behaviour of *Bemisia tabaci*".
11) African Cassava Whitefly Project-Phase II (2019-2022) proposal narrative. John Colvin, NRI. Internal/Personal communication.
12) Taylor NJ (2012). GM Crops & Foods, 3(2): 93-103. "The VIRCA project".
13) Hillocks (2003). Int J Pest Manag, 49(3): 225-234. "Cassava brown streak disease: a review of present knowledge and research needs".
14) Legg JP (2015). Adv. Vir Res, 91: 85-142. "Cassava virus diseases: biology, epidemiology and management".
15) Kawuki RS (2016). Breeding Sci, 66: 560-571. "Eleven years of breeding efforts to combat cassava brown streak disease".
16) Hahn SK (1980). Euphytica, 29: 673-683. "Breeding cassava for resistance to cassava mosaic disease".
17) Fregene M (2000). Theor Appl Genet, 100: 678-685. "AFLP analysis of African cassava (*Manihot esculenta* Crantz) germplasm resistant to the cassava mosaic disease (CMD)".
18) https://www.newvision.co.ug/news/1318718/cassava-varieties-farmers-hope
19) Adriko J (2011). Afr J Agirc Res, 6(3): 521-531. "Response of improved cassava varieties in Uganda to cassava mosaic disease (CMD) and their inherent resistance mechanisms".
20) http://www.fao.org/3/a0154e/A0154E11.htm
21) Mukiibi DR (2019). Crop Prot, 115: 104-112."Resistance of advanced cassava breeding clones to infection by major viruses in Uganda".
22) Crop Improvement, adoption and impact of improved varieties in food crops in Sub-Saharan Africa. Edited by Thomas S. Walker and Jeffrey Alwang. CGIAR and CABI publications. 2015. ISBN 978-1-78064-401-1
23) Cassava: Biology, production and utilization. Edited by RJ Hillocks, JM Thresh, AC Bellotti. CABI Publications, Wallingford, UK. 2002. ISBN 0-85199-524-1.
24) Ozimati A (2019). Crop Sci, 59: 460-473. *“Genetic variation and trait correlations in an East African cassava breeding population for genomic selection”*.
